# Hormone use among the transgender, transvestites and non-binary population of Porto Alegre, Brazil, 2021: a cross-sectional study

**DOI:** 10.1590/S2237-96222024v33e2024335.especial.en

**Published:** 2024-12-16

**Authors:** Lara Colles de Oliva Araujo, Luciane Kopittke, Vinicius Vicari

**Affiliations:** 1 Fundação Oswaldo Cruz, projeto A Hora é Agora, Porto Alegre, RS, Brazil; 2 Hospital Nossa Senhora da Conceição, Escola GHC, Porto Alegre, RS, Brazil; 3 Hospital Nossa Senhora da Conceição, Gerência de Atenção Primária à Saúde, Porto Alegre, RS, Brazil

**Keywords:** Identidad de Género, Personas Transgénero, Hormonas, Acceso a los Servicios de Salud, Estudios Transversales, Gender Identity, Transgender People, Hormones, Access to Health Services, Cross-Sectional Studies

## Abstract

**Objective:**

To describe the prevalence of hormone use, sociodemographic profile, and access to health services among the transgender, transvestite, and nonbinary population in Porto Alegre, capital city of Rio Grande do Sul state.

**Methods:**

A cross-sectional study was conducted between September and November 2021, using a snowball sampling method (n=65). Data were collected through a self-administered questionnaire with transgender people aged 18 years or older.

**Results:**

High hormone use was observed (n=47), with a higher occurrence of self-medication among transgender women, transvestites, and transfeminine people (n=10). Health monitoring predominantly occurs in gender identity outpatient clinics (n=56). Episodes of transphobia when accessing health services were reported by 28 individuals.

**Conclusion:**

This study demonstrated high prevalence of hormone use, highlighted specific patterns of use across different gender identities, and the need for improvements in access to healthcare services.

## Study contributions

**Main results:**

High prevalence of hormone use was observed (n=47), with emphasis on formulations containing estrogen (n=12) or testosterone cypionate (n=22). Self-administered hormone therapy was higher among transgender women, transvestites and transfeminine people (n=10).

**Implications for services:**

This study contributes to understanding the process of self-administered hormone therapy and the need for appropriate interventions. It highlights the importance of establishing strategies that ensure access without discrimination based on gender identity.

**Perspectives:**

Further studies are needed on this topic to encourage the inclusion of medications used in hormone therapy in the National List of Essential Medicines, with the aim of making access to hormones more equitable and universal.

## RESUMEN

**Objetivo:**

Describir la prevalencia del uso de hormonas, el perfil sociodemográfico y el acceso a los servicios de salud por parte de la población trans, travesti y no binaria en Porto Alegre, Rio Grande do Sul.

**Métodos:**

Se utilizó un estudio transversal, entre septiembre y noviembre de 2021, con muestreo por “bola de nieve” (n=65), realizado a partir de un cuestionario autoadministrado con personas trans de 18 años o más.

**Resultados:**

Se observó un alto uso de hormonas (n=47), con mayor incidencia de automedicación hormonal entre mujeres trans, travestis y personas transfemeninas (n=10). El seguimiento en salud se realiza principalmente en ambulatorios de identidad de género (n=56). Episodios de transfobia y travestifobia al acceder a los servicios de salud fueron reportados por 28 personas.

**Conclusión:**

Este estudio demostró el alto uso de hormonas, las especificidades en su utilización entre las diferentes identidades de género y la necesidad de mejorar el acceso a los servicios de salud.

## INTRODUCTION

Hormone therapy is one of the most commonly used technologies among transgender people, transvestite and non-binary people in their process of embodiment. This practice represents a possibility of autonomy and construction of bodies, subjectivities and the expression of identities.^1-3^ The body, as an interface between the social and individual, plays an important role in the process of subjectivation and recognition of new gender markers, which are also constructed through the use of hormones.^4,5^

Hormone therapy is associated with improved self-esteem, quality of life, the promotion of greater well-being and personal and social acceptance.^6,7^ Difficulty in accessing health services and care technologies leads many transgender people to start hormone therapy without a medical prescription or professional guidance.^8^ The self-medication process highlights the precarious nature of healthcare assistance for this population, characterized by the denial of their public existence, stigmatization, and discrimination.^9^

In order to reduce access barriers and the fragility of healthcare service for this population, specific healthcare services for the transgender population have been established in Brazil, such as gender identity outpatient clinics. In Porto Alegre, capital city of Rio Grande do Sul state, the following services comprise the healthcare network: the T Outpatient Clinic of the Municipal Health Department, Gender Identity Outpatient Clinic of the Grupo Hospitalar Conceição and the Gender Identity Program at the Hospital de Clínicas de Porto Alegre.

Despite the widespread use of hormones among the transgender population, there is limited research in Brazil that analyzes the pattern of this practice and the occurrence of discrimination in accessing health services.

This research aimed to describe the prevalence of hormone use, the sociodemographic profile and access to health services among the transgender, transvestite and non-binary population in Porto Alegre. This article adopts a non-pathologizing perspective of transsexuality and intends to discuss the medication as one of the elements of health promotion.

## METHODS

This was a cross-sectional, multicenter study, conducted in two gender identity outpatient clinics in Porto Alegre, with data collection carried out from September to November 2021. The inclusion criteria for the study were: self- identification as a transgender person, transvestite or non-binary person, aged 18 years or older and residing in Porto Alegre.

Given the social marginalization experienced by the transgender population, snowball sampling was employed as it is a non-probabilistic technique used when the population of interest is difficult to reach or identify. Instead of selecting samples randomly, this technique starts with a small group of participants, who then nominate other participants for the study, causing the sample to grow as new participants are incorporated.^10^

In this study, three initial key informants, also called seeds, were considered. These included a trans activist from Porto Alegre and two professionals who worked in gender identity outpatient clinics. The seeds were chosen due to their proximity to the transgender population and the possibility of working during the COVID-19 pandemic. Dissemination occurred both in person and virtually, with WhatsApp being the main dissemination tool used by the seeds, due to the pandemic context. Through this application, the questionnaire was sent to individuals receiving care at the outpatient clinics by the seeds who worked in these services and forwarded by the trans activist to the community groups for transgender people in the city. Invitations to participate in the study was sent via WhatsApp up to three times, with a one-week interval between attempts, in cases where the individual did not respond to the first two contacts.

Data were collected via a self-administered questionnaire filled out by participants using an online form through Google Docs. The questionnaire could only be completed after participants had read and agreed to the terms of the Free and Informed Consent Form.

The variables included in the questionnaire were: gender identity, sexual orientation, age group, race/skin color, schooling, occupation, income, types of healthcare services accessed, frequency of access, episodes of transphobia in the services accessed, type of hormone used among those undergoing hormone therapy, route of administration, method of acquisition and the presence of adverse events related to hormone use.

The analysis of the variables was performed by calculating simple frequencies in order to describe the characteristics of the sample. Statistical analyses were performed using the Statistical Package for the Social Sciences , version 23.0.

The research project was developed in accordance with the current regulations expressed in Resolutions No. 466/2012 and No. 510/2016 of the National Health Council. The project was approved by the GHC Research Ethics Committee in September 2021 (CAAE: 43299521.0.0000.5530). The confidentiality of data and information that could identify participants was ensured, in compliance with the General Data Protection Law (Law No. 13,709, of August 14, 2018).

## RESULTS

The survey was answered by 68 participants, with subsequent exclusion of three people for not meeting the study’s inclusion criteria. Of the remaining participants, 33 identified as transgender men and 19 identified as transgender women. Twenty-four people identified as heterosexual; 18,pansexual; and 15 as bisexual.

The sample predominantly comprised people aged 18 and 24 years (n=30), self-declared as white (n=49), with most having completed high school (n=45) and holding some employment status, either on the books (n=23), as self-employed workers (n=14), or off the books (n=12). An equal proportion (n=29) was found among people with a monthly income of less than 1 minimum wage or between 1 and 3 minimum wages, with a small portion earning between 3 and 5 minimum wages (n=7) (Table 1).

**Table 1:** Sociodemographic profile of the study participants, Porto Alegre, Brazil, 2021 (n=65)

Variables	n
**Gender identity**	
Transvestite	3
Transgender woman	19
Transgender man	33
Non-binary	9
Gender fluid	1
**Sexual orientation**	
Pansexual	18
Bisexual	15
Homosexual	7
Heterosexual	24
Asexual	1
**Age group (years)**	
18-24	30
25-34	26
35-41	9
**Race/skin color**	
White	49
Black	9
Mixed-race	6
Indigenous	1
**Education level**	
Without education	0
Elementary education	9
High school	45
Higher education	9
Postgraduate degree	2
**Occupation**	
Works on the books	23
Works off the books	12
Self-employed	14
Student	6
Unemployed	10
**Income (minimum wage)**	
Less than 1	29
Between 1-3	29
Between 3-5	7

All participants reported seeking healthcare services, primarily through the Brazilian National Health System (*Sistema Único de Saúde* – SUS) (n=60), on an occasional basis (n=40), with the majority receiving care in gender identity outpatient clinics (n=56) and in primary healthcare centers (n=28). Episodes of transphobia were reported by 28 individuals when seeking healthcare services (Table 2).

**Table 2:** Information related to health services accessed by transgender people, Porto Alegre, Brazil, 2021 (n= 65)

Variables	n
**Types of health services accessed**	
Brazilian National Health System	60
Private sector	14
Health insurance	19
**Frequency of accessing services**	
Always	21
Occasionally	40
In emergencies	5
**Services sought for care**	
Primary healthcare center	28
Gender identity outpatient clinic	56
Emergency room	14
Mental health services	3
Hospitals	21
Private medical clinics	21
**Incidents of transphobia in the services accessed**	
Yes	28
No	37

Table 3 provides information related to hormone therapy among individuals who reported using hormones (n=47). For transgender women, transvestites, and transfeminine individuals, the most commonly used medications were those containing estrogen (n=12) or antiandrogens (n=11). The primary route of administration was oral (n=13). Medications were mainly purchased without a medical prescription at a pharmacy (n=10). For transgender men or transmasculine individuals, the most commonly used hormone was testosterone cypionate (n=22). The main route of administration was injectable (n=25). These medications were mainly obtained from pharmacies with a medical prescription (n=25). It could be seen that transgender women, transvestites, and transfeminine individuals reported a higher frequency (n=6) of adverse effects from hormone use.

**Table 3:** Information related to hormone use, Porto Alegre, Brazil 2021

Variables	Self-reported gender identities	
	Transgender women, transvestites and transfeminine people	Transgender men and transmasculine people
	(n=20)	(n=27)
**Type of hormone used**		
Estrogen	12	-
Progesterone	0	-
Estrogen and progesterone	7	-
Antiandrogen	11	-
Testosterone undecanoate	-	4
Testosterone undecylate	-	2
Testosterone cypionate	-	22
Testosterone gel 1% or 5%	-	2
Testosterone propionate, fempropionate, isocaproate and decanoate	-	1
**Route of administration**		
Injectable	8	25
Oral	13	0
Topical	2	2
**Form of acquisition**		
Pharmacy with prescription	10	2
Pharmacy without prescription	15	25
Other transgender people	1	1
Internet	3	1
**Adverse event due to hormone use**		
Yes	6	6
No	8	15
Does not know	6	6

## DISCUSSION

A high prevalence of hormone use was observed among the interviewees, regardless of gender identity. These findings align with previous studies that reported high rates of hormone use among individuals with different gender identities, aimed at promoting bodily modifications.^11-14^

The use of hormones by the transgender population can be understood as an attempt to shape identity according to their own conceptions of gender, within the rigid and normative structure in which gender is situated, in pursuit of a socially comprehensible existence.^15^ It is crucial to understand that corporeality transcends the notion of body and self-satisfaction. This may represent a social self-protection mechanism, as meeting cisnormative standards may prevent social embarrassment and transphobic violence.^16^

Although these substances are not clinically approved by the Brazilian Health Regulatory Agency for the specific purpose of hormone therapy, the practice is supported by the Ministry of Health and the Brazilian Federal Council of Medicine.^17^ The lack of regulation concerning the use of these hormones prevents their distribution to SUS users, fostering self-administration and increasing the associated risks.

In a study conducted in the United States on hormone therapy among transgender people, it was found that 9.17% used hormones without a prescription. This practice highlights one of the mechanisms used by these people to avoid potential situations of discrimination or violence when seeking prescriptions from healthcare professionals.^18^ Given that hormone therapy is an important part of the health and well-being of transgender people, it is essential to make it a safe strategy, avoiding the negative impacts associated with its unsupervised and unsafe use.^3^ Self-medication rates can be used to analyze the precariousness of healthcare provided to the transgender population and the lack of access to health services and supplies.^9,19^

In this study, the number of people who reported adverse effects from hormone use was relatively low. A high level of lack of knowledge about the adverse effects of hormone use, was observed. This highlights the need for these people to be monitored by healthcare professionals.

This study sample consisted predominantly of young adults, with the majority being under 35 years of age. This finding is in line with previous studies, in which a young population sample with an average age of under 35 years, was observed.^20-22^ It is worth noting that, given the life expectancy of transgender people in Brazil, which averages 35 years,^23^ the focus on narrow age ranges is a persistent issue.

The majority of participants in this study self-identified as white. Studies conducted in other regions of Brazil reported different race/skin color data, with prevalence of individuals who self-identified as mixed-race^12,20,22^ or mixed-race and Black,^13^ which reflects the different territorial divisions and colonization process in Brazil.

Participants in this study had high education level. Although the study did not investigate the reasons behind this finding, the recruitment method and data collection process may have introduced selection bias, due to the population’s level of education and access to technology. This excludes illiterate individuals who do not feel confident reading and filling out online forms. Given that early participants recruited subsequent ones, this is reflected in the similar characteristics regarding the sociocultural level. Data obtained in this study may be the result of policies promoting access and retention in educational settings, such as the use of the social name and the implementation of affirmative actions for transgender people in higher education.^24,25^

The identity profile of the interviewees was primarily concentrated among transgender men and transgender women, followed by a smaller proportion of non-binary people and transvestites. The lower frequency of transvestites may be a limitation of this study, taking into consideration the selection method used, in addition to reflecting a long process of marginalization and discrimination in healthcare services. This leads to greater barriers to access and difficulties in remaining in these services.^26^

Access to the labor market by transgender people is an increasingly discussed issue due to the difficulties they face in securing and maintaining employment.^27^ In this study, it was observed that nearly half (n=26) of the participants worked off the books or were self-employed. In a study conducted in the Federal District, 12.6% of transgender women and transvestites worked on the books, with a significant proportion declaring themselves as self-employed (58.7%).^12^

Although Primary Health Care (PHC) is considered the entry point to the SUS, the most accessed health service among participants in this study were the gender identity outpatient clinics. This fact can be explained by the numerous forms of violence experienced by transgender people when accessing PHC, such as stigmatization, discrimination, disrespect for their social name, lack of professional training and inadequate care.^28^

Based on data obtained, it was possible to observe a high rate of hormone use among the study population, regardless of gender identity, with self-administration being more common among transgender women, transvestites and transfeminine people. As hormone therapy is a process linked to body modification and self-subjectivation, it underscores the importance of healthcare professionals providing guidance on hormone use and potential adverse effects. Understanding hormone use patterns helps in the development of prescription and harm reduction strategies. The importance of conducting research with a greater number and more diverse populations to establish the needs of various subgroups within the transgender population is highlighted.

It is crucial to promote the inclusion and implementation of public policies aimed at reducing barriers to accessing hormones. Including the medications used for hormone therapy in the National List of Essential Medicines is necessary to optimize healthcare, with the aim of providing truly universal and equitable access to health services and supplies for transgender people.
