# Supplementary material for: Hormone use among the transgender, transvestites and non-binary population of Porto Alegre, Brazil, 2021: a cross-sectional study
Source: Epidemiol Serv Saude. 2024 Dec 16;33(spe1):e2024335. doi: 10.1590/S2237-96222024v33e2024335.especial.en (PMC11654815; doi:10.1590/S2237-96222024v33e2024335.especial.en)
Supplement: Translation

# Translation: Utilização de hormônios pela população trans, travesti e não-binária de Porto Alegre em 2021: um estudo transversal

**DOI:** 10.1590/S2237-96222024v33e2024335.especial.pt

**Authors:** Lara Colles de Oliva Araujo, Luciane Kopittke, Vinicius Vicari

**Affiliations:** 1 Fundação Oswaldo Cruz, projeto A Hora é Agora, Porto Alegre, RS, Brasil; 2 Hospital Nossa Senhora da Conceição, Escola GHC, Porto Alegre, RS, Brasil; 3 Hospital Nossa Senhora da Conceição, Gerência de Atenção Primária à Saúde, Porto Alegre, RS, Brasil

**Keywords:** Identidade de Gênero, Pessoas Transgênero, Hormônios, Acesso aos Serviços de Saúde, Estudos Transversais

## Abstract

**Objetivo:**

Descrever a prevalência do uso de hormônios, o perfil sociodemográfico e o acesso aos serviços de saúde pela população trans, travesti e não-binária em Porto Alegre, no Rio Grande do Sul.

**Métodos:**

Utilizou-se estudo transversal, entre setembro e novembro de 2021, com amostragem por “bola de neve” (n=65), realizado a partir de questionário autoaplicável com pessoas trans de idade igual ou superior a 18 anos.

**Resultados:**

Verificou-se o alto uso de hormônios (n=47), com maior ocorrência de auto-hormonização entre as mulheres trans, travestis e pessoas transfemininas (n=10). O acompanhamento em saúde ocorre principalmente nos ambulatórios de identidade de gênero (n=56). Episódios de transfobia e travestifobia ao acessar os serviços de saúde foram relatados por 28 pessoas.

**Conclusão:**

Este estudo demonstrou o alto uso de hormônios, as especificidades na utilização entre as diferentes identidades de gênero e a necessidade de melhorias no acesso aos serviços de saúde.

## Contribuições do estudo

**Principais resultados:**

Verificou-se o alto uso de hormônios (n=47), com destaque para as formulações contendo estrogênio (n=12) ou cipionato de testosterona (n=22). A auto-hormonização foi maior entre as mulheres trans, travestis e pessoas transfemininas (n=10).

**Implicações para os serviços:**

O estudo contribui no entendimento do processo de auto-hormonização e intervenções adequadas. Este estudo aponta a importância do estabelecimento de estratégias que garantam o acesso sem discriminação relacionada à identidade de gênero.

**Perspectivas:**

Fazem-se necessários mais estudos na área para fomentar a inclusão dos medicamentos utilizados na hormonização na Relação Nacional de Medicamentos Essenciais, com a finalidade de tornar o acesso aos hormônios mais equânime e universal.

## INTRODUÇÃO

O uso de hormônios é uma das tecnologias mais utilizadas pelas pessoas trans, travestis e não-binárias nos processos de corporificação. Tal prática representa uma possibilidade de autonomia e construção dos corpos, das subjetividades e da expressão das identidades.^1-3^ O corpo, enquanto interface entre o social e o individual, exerce uma importante função no processo de subjetivação e de reconhecimento dos novos signos de gênero que vão sendo construídos também através da utilização de hormônios.^4,5^

A hormonização está associada à melhora da autoestima, qualidade de vida, promoção de maior bem-estar e aceitação pessoal e social.^6,7^ A dificuldade de acesso aos serviços de saúde e tecnologias de cuidado faz com que muitas pessoas trans iniciem a hormonização sem prescrição médica ou orientação profissional.^8^ O processo de automedicação explicita a precariedade da assistência a essa população caracterizada pela negação da sua existência pública, pela estigmatização e pela discriminação.^9^

Com o intuito de reduzir as barreiras de acesso e a fragilidade assistencial à saúde dessa população, serviços específicos de assistência à saúde para população trans foram instituídos no Brasil, como os ambulatórios de identidade de gênero. Em Porto Alegre, no Rio Grande do Sul, os seguintes serviços compõem a rede de assistência: Ambulatório T da Secretária Municipal de Saúde, Ambulatório de Identidade de Gênero do Grupo Hospitalar Conceição e o Programa de Identidade de Gênero do Hospital de Clínicas de Porto Alegre. 

Apesar do uso difundido de hormônios entre a população trans, existem poucas pesquisas no Brasil que analisam o padrão dessa prática e a ocorrência de discriminação no acesso aos serviços de saúde.

Esta pesquisa objetivou descrever a prevalência do uso de hormônios, o perfil sociodemográfico e o acesso aos serviços de saúde pela população trans, travesti e não-binária em Porto Alegre. Este artigo parte de um ponto de vista não patologizante da transexualidade, portanto pretende-se discutir o medicamento como um dos elementos de promoção à saúde.

## MÉTODOS

Tratou-se de estudo transversal e multicêntrico, realizado em dois ambulatórios de identidade de gênero de Porto Alegre, com coleta de dados realizada de setembro a novembro de 2021. Os critérios de inclusão para o estudo foram: identificar-se enquanto pessoa trans, travesti ou não-binária, possuir idade igual ou superior a 18 anos e residir em Porto Alegre. 

Considerando a marginalização social experienciada pela população trans, a amostragem por “bola de neve” foi empregada por ser uma técnica não probabilística utilizada em situações em que a população de interesse é de difícil acesso ou identificação. Em vez de selecionar amostras de forma aleatória, essa técnica começa com um pequeno grupo de participantes. Estes então indicam outros participantes para o estudo, e a amostra cresce à medida que novos participantes são incorporados.^10^

Neste estudo, foram considerados três informantes chaves iniciais, também chamados de sementes. Estes foram uma ativista trans de Porto Alegre e dois profissionais que atuavam nos ambulatórios de identidade de gênero. As sementes foram escolhidas devido à proximidade com a população trans e pelas possibilidades de trabalho em meio à pandemia da covid-19. A divulgação ocorreu de forma presencial e virtual, sendo o WhatsApp a principal ferramenta de divulgação pelos indivíduos sementes, devido ao contexto pandêmico. Através desse aplicativo, o questionário foi enviado para as pessoas usuárias dos ambulatórios pelas sementes que atuavam nesses serviços e encaminhado pela ativista trans para os grupos de convivência de pessoas trans do município. O convite para participar da pesquisa foi enviado via WhatsApp até três vezes, com um intervalo de uma semana entre as tentativas, caso a pessoa não respondesse aos dois primeiros contatos.

Os dados foram coletados por meio das respostas do questionário autoaplicável preenchido pelas participantes através de um formulário no editor de documentos *on-line* Google Docs. O questionário só pôde ser respondido após aceite da participação no estudo por meio da leitura e concordância com o Termo de Consentimento Livre e Esclarecido. 

As variáveis que compuseram o questionário foram: identidade de gênero, orientação sexual, faixa etária, raça/cor da pele, escolaridade, ocupação, renda, tipos de serviços de saúde acessados, frequência de acesso, episódios de transfobia e travestifobia nos serviços acessados, tipo de hormônio utilizado entre as pessoas que realizam hormonização, via de administração, forma de aquisição e presença de eventos adversos pelo uso de hormônios. 

A análise das variáveis foi realizada através do cálculo de frequências simples com o intuito de descrever as características da amostra. As análises estatísticas foram realizadas utilizando o aplicativo Statistical Package for the Social Sciences, versão 23.0. 

O projeto de pesquisa foi desenvolvido em conformidade com as normas vigentes expressas nas Resoluções nº 466/2012 e nº 510/2016 do Conselho Nacional de Saúde. O projeto foi aprovado pelo Comitê de Ética em Pesquisa do GHC em setembro de 2021 (CAAE: 43299521.0.0000.5530). Foi assegurada a confidencialidade dos dados e das informações que possibilitem a identificação de participantes, seguindo as regras da Lei Geral de Proteção de Dados Pessoais (Lei nº 13.709, de 14 de agosto de 2018).

## RESULTADOS

A pesquisa foi respondida por 68 participantes, com posterior exclusão de 3 pessoas por não preencherem os critérios de inclusão do estudo. Destes, 33 participantes identificavam-se como homens trans, e 19, como mulheres trans. Vinte e quatro pessoas se declararam como heterossexuais; 18, como pansexuais; e 15, como bissexuais. 

A amostra foi composta majoritariamente por pessoas entre 18 e 24 anos (n=30), autodeclaradas como brancas (n=49), com escolaridade concentrada entre aquelas que possuem ensino médio completo (n=45) e com algum tipo de vínculo empregatício, seja em trabalhos formais com carteira assinada (n=23), seja como autônomos (n=14), ou sem carteira assinada (n=12). Verificou-se a mesma proporção (n=29) entre as pessoas com renda mensal menor que 1 salário mínimo ou entre 1 e 3 salários mínimos, com uma pequena parcela entre 3 e 5 salários mínimos (n=7) (Tabela 1).

**Tabela 1:** Perfil sociodemográfico das pessoas participantes da pesquisa, Porto Alegre, 2021 (n=65)

Variáveis	n
**Identidade de gênero**	
Travesti	3
Mulher trans	19
Homem trans	33
Pessoa não-binária	9
Gênero fluido	1
**Orientação sexual**	
Pansexual	18
Bissexual	15
Homossexual	7
Heterossexual	24
Assexual	1
**Faixa etária (anos)**	
18-24	30
25-34	26
35-41	9
**Raça/cor de pele**	
Branca	49
Preta	9
Parda	6
Indígena	1
**Escolaridade**	
Sem escolaridade	0
Ensino fundamental	9
Ensino médio	45
Ensino superior	9
Pós-graduação	2
**Ocupação**	
Empregado com carteira assinada	23
Empregado sem carteira assinada	12
Autônomo	14
Estudante	6
Desempregado	10
**Renda (salário mínimo)**	
Menor que 1	29
Entre 1-3	29
Entre 3-5	7

Todas as pessoas afirmaram buscar por serviços de saúde, principalmente no Sistema Único de Saúde (SUS) (n=60), de forma eventual (n=40), com atendimento majoritariamente realizado nos ambulatórios de identidade de gênero (n=56) e nas unidades básicas de saúde (n=28). Episódios de transfobia e travestifobia foram relatados por 28 pessoas ao buscarem os serviços de saúde (Tabela 2).

**Tabela 2:** Informações relacionadas aos serviços de saúde acessados pelas pessoas trans, Porto Alegre, 2021 (n=65)

Variáveis	n
**Tipos de serviços de saúde acessados**	
Sistema Único de Saúde	60
Rede privada	14
Plano de saúde	19
**Frequência de acesso aos serviços**	
Sempre	21
Eventualmente	40
Em emergências	5
**Serviços em que busca atendimento**	
Unidade básica de saúde	28
Ambulatório de identidade de gênero	56
Unidade de pronto atendimento	14
Serviços de saúde mental	3
Hospitais	21
Consultório particular	21
**Episódios de trans/travestifobia nos serviços acessados**	
Sim	28
Não	37

A Tabela 3 traz as informações relacionadas à hormonização entre as pessoas que referiram fazer o uso de hormônios (n=47). Para mulheres trans, travestis e pessoas transfemininas, o medicamento mais utilizado foi o que possui estrogênio (n=12) ou antiandrogênico (n=11) na formulação. A principal via de administração foi a oral (n=13). A aquisição dos medicamentos ocorreu principalmente através da compra sem receita médica em farmácia (n=10). Para homens trans ou pessoas transmasculinas, o hormônio mais utilizado foi o cipionato de testosterona (n=22). A principal via de administração foi a injetável (n=25). A aquisição ocorreu principalmente em farmácia com receit**a** médica (n=25). Verificou-se que as mulheres trans, travestis e pessoas transfemininas relataram maior frequência (n=6) de efeitos indesejados causados pelo uso de hormônios. 

**Tabela 3:** Informações relacionadas ao uso de hormônios, Porto Alegre, 2021

Variáveis	Identidades de gênero autorrelatadas	
	Mulheres trans, travestis e pessoas transfemininas	Homens trans e pessoas transmasculinas
	(n=20)	(n=27)
**Tipo de hormônio utilizado**		
Estrogênio	12	-
Progesterona	0	-
Estrogênio e progesterona	7	-
Antiandrogênico	11	-
Undecanoato de testosterona	-	4
Undecilato de testosterona	-	2
Cipionato de testosterona	-	22
Testosterona gel 1% ou 5%	-	2
Propionato, fempropionato, isocaproato e decanoato de testosterona	-	1
**Via de administração**		
Injetável	8	25
Oral	13	0
Tópico	2	2
**Forma de aquisição**		
Farmácia sem receita	10	2
Farmácia com receita	15	25
Outras pessoas trans	1	1
Internet	3	1
**Presença de evento adverso pelo uso de hormônio**		
Sim	6	6
Não	8	15
Não sabe	6	6

## DISCUSSÃO

Observou-se alta ocorrência do uso de hormônios entre as pessoas entrevistadas independentemente da identidade de gênero. Esses resultados estão de acordo com os estudos previamente realizados em que foi observada a alta taxa de hormonização, entre as pessoas com diferentes identidades de gênero analisadas, para promover modificações na corporalidade dos sujeitos.^11-14^

A utilização de hormônios pela população trans pode ser entendida como a tentativa de moldar a identidade de acordo com suas próprias concepções de gênero, na estrutura normativa e altamente rígida em que o gênero se situa, para a busca da existência socialmente compreendida.^15^ É importante ter o entendimento de que a corporeidade transcende a noção de corpo e autossatisfação. Isso pode representar um dispositivo de autoproteção social, uma vez que atender aos padrões cisnormativos pode evitar constrangimentos sociais e violências transfóbicas.^16^

Apesar dessas substâncias não serem protocoladas clinicamente pela Agência Nacional de Vigilância Sanitária para o fim específico da hormonização, a prática é amparada pelo Ministério da Saúde e pelo Conselho Federal de Medicina.^17^ A falta de regulamentação do uso desses hormônios impede a garantia da sua distribuição para usuários do SUS. Isso favorece a auto-hormonização e aumenta os riscos associados a tal prática. 

Em estudo realizado nos Estados Unidos sobre uso de hormônios pelas pessoas trans, foi verificado que 9,17% utilizam hormônios sem prescrição. Essa prática demonstra um dos mecanismos utilizados por essas pessoas para evitar possíveis situações de discriminação ou violência durante os atendimentos para a obtenção da prescrição com profissionais de saúde.^18^ Visto que a hormonização é parte importante do processo de saúde e do bem-estar das pessoas trans, é preciso torná-la uma estratégia segura, evitando que ela se caracterize pelos impactos negativos do seu uso sem orientação e segurança.^3^ Pode-se utilizar as taxas de automedicação para analisar a precariedade da assistência destinada à população trans e a falta de acesso a serviços e insumos de saúde.^9,19^

Neste estudo, o índice de pessoas que referiram ter vivenciado efeitos adversos provenientes do uso de hormônios foi relativamente baixo. Verificou-se alto índice da falta de conhecimento de quais seriam os efeitos adversos provenientes do uso de hormônios. Esse dado reforça a necessidade de o acompanhamento em saúde dessas pessoas ser realizado por um profissional da saúde. 

O estudo contou com a amostra de adultos jovens, com ampla maioria abaixo de 35 anos. Esse dado está de acordo com estudos realizados anteriormente, nos quais se observou uma amostra populacional jovem, com idade média abaixo dos 35 anos.^20-22^ Ressalta-se que, tratando-se de uma população cuja expectativa média de vida no Brasil é de 35 anos,^23^ a possibilidade de abranger restritas faixas etárias é uma constante. 

Na amostra, a maior ocorrência é de pessoas que se autodeclararam brancas. Estudos conduzidos em outras regiões do Brasil trouxeram dados de raça/cor de pele distintos, com prevalência de pessoas autodeclaradas enquanto pardas^12,20,22^ ou pardas e pretas,^13^ o que reflete os diferentes recortes territoriais e processos de colonização no Brasil. 

Os participantes deste estudo apresentaram alta escolaridade. Apesar de o estudo não ter investigado o motivo desse dado, o método escolhido para o recrutamento dos participantes e a coleta de dados pode ter potencializado a ocorrência de vieses de seleção em decorrência da escolaridade da população e do acesso à tecnologia. Isso exclui indivíduos não alfabetizados e que não se sentem seguros com a leitura e o preenchimento de documentos *on-line*. Como os primeiros participantes recrutavam os posteriores, tem-se esse reflexo nas características semelhantes entre o nível sociocultural. O dado obtido neste estudo pode ser resultado de políticas de acesso e permanência no ambiente de ensino, como o uso do nome social e a efetivação de ações afirmativas para pessoas trans no ingresso ao ensino superior.^24,25^

O perfil identitário das pessoas entrevistadas concentra-se, principalmente, entre homens trans e mulheres trans seguido pela menor parcela de pessoas não-binárias e travestis. A menor frequência de travestis pode ser uma limitação do estudo, considerando o método de seleção utilizado, além do reflexo de um longo processo de marginalização e discriminação nos serviços de saúde. Isso culmina em maiores barreiras de acesso e dificuldade de permanência nesses serviços.^26^

O acesso ao mercado de trabalho pelas pessoas trans é um assunto cada vez mais abordado em decorrência das dificuldades enfrentadas desde a sua inserção até a permanência nos empregos.^27^ Neste estudo, observou-se que quase metade (n=26) das pessoas trabalhavam sem carteira assinada ou de forma autônoma. Em um estudo realizado no Distrito Federal, verificou-se que 12,6% das mulheres trans e travestis estavam empregadas no mercado formal, e grande parte se declarava autônoma (58,7%).^12^

Apesar de a Atenção Primária à Saúde (APS) se configurar como porta de entrada do SUS, o serviço de saúde mais acessado entre participantes deste estudo foram os ambulatórios de identidade de gênero. Esse fato pode ser explicado devido às inúmeras violências vivenciadas pelas pessoas trans ao acessarem a APS, como estigmatização, discriminação, desrespeito ao nome social, falta de capacitação profissional e atendimento inadequado.^28^

A partir dos dados obtidos, foi possível observar o alto índice de hormonização entre a população do estudo, independentemente da identidade de gênero, sendo a auto-hormonização uma prática mais recorrente entre as mulheres trans, travestis e pessoas transfemininas. Uma vez que a hormonização se configura como um processo ligado à construção corporal e de modos de subjetivação de si, nota-se a importância da orientação sobre o uso de hormônios e seus possíveis efeitos adversos por parte de profissionais de saúde. O entendimento sobre os padrões de uso de hormônios auxilia na elaboração de estratégias de prescrição e redução de danos. É ressaltada a importância da realização de pesquisas com maior número e diversidade de sujeitos para estabelecer as necessidades dos vários subgrupos na população trans. 

Salienta-se a relevância de fomentar a inclusão e efetivação de políticas públicas que trabalhem em prol da redução das barreiras de acesso aos hormônios. A inclusão dos medicamentos utilizados na hormonização na Relação Nacional de Medicamentos Essenciais é uma necessidade para a otimização do cuidado em saúde, com o intuito de prover o acesso aos serviços e insumos de saúde de fato mais universal e equânime às pessoas trans.
